# Activity of dose-dense outpatient intravenous Interleukin-2 preceded by famotidine in metastatic clear cell cancer of the kidney

**DOI:** 10.1186/2051-1426-1-S1-P29

**Published:** 2013-11-07

**Authors:** Walter Quan, Francine M Quan

**Affiliations:** 1 East Carolina University, Greenville, NC, USA; 2 Loma Linda University, Loma Linda, CA, USA

## 

Daily short intravenous infusions of Interleukin-2 (IL-2) have been developed to decrease toxicity while maintaining anticancer activity of this agent against kidney cancer. Such IL-2 schedules have previously been shown to increase Lymphokine Activated Killer cell (LAK) numbers (Quan) and LAK activity (Mitchell). Famotidine may increase LAK activity by increasing Interleukin-2 internalization by the IL-2 receptor on lymphocytes (Tsunoda). We treated 15 patients with metastatic clear cell kidney cancer using IL-2 18 Million IU/M2 intravenously over 15-30 minutes preceded by famotidine 20 mg IV daily for 3 days for 6 consecutive weeks on an outpatient basis. Cycles were repeated every 8 weeks until disease progression. Patient characteristics: 7 males/8 females, median age-59 (range: 28-70), median ECOG performance status-1; common metastatic sites: lungs (14), lymph nodes (9), liver (4), bone (4), and pancreas (4). Prior systemic therapy: oral TKI (8), Interleukin-2 (6), mTor inhibitor (2), no prior (3). Prior nephrectomy (9). Most common toxicities were rigors (13), arthralgia/myalgia (12), nausea/emesis (11), fever (11), hypotension (11), and constipation (8). All episodes of hypotension were transient and alleviated with outpatient intravenous fluid. No patients have required hospitalization due to toxicity of therapy. There have been no treatment-related deaths. One complete response (7%) (in lungs lasting 13 months) and four partial responses (26%) have been seen (total response rate =33%; 95% CI:15-59%). Responses have occurred in lungs, liver, lymph nodes, and bone. Median response duration = 5 months (2-13). Median survival = 13.75 months (3-41 months). Two patients are alive at 35.7+ and 35.2+ months. Dose-dense outpatient intravenous Interleukin-2 preceded by famotidine has activity in metastatic clear cell kidney cancer.

